# CABG Surgery Remains the best Option for Patients with Left Main Coronary Disease in Comparison with PCI-DES: Meta-Analysis of Randomized Controlled Trials

**DOI:** 10.21470/1678-9741-2017-0081

**Published:** 2017

**Authors:** Michel Pompeu Barros Oliveira Sá, Artur Freire Soares, Rodrigo Gusmão Albuquerque Miranda, Mayara Lopes Araújo, Alexandre Motta Menezes, Frederico Pires Vasconcelos Silva, Ricardo Carvalho Lima

**Affiliations:** 1 Division of Cardiovascular Surgery, Pronto-Socorro Cardiológico de Pernambuco (PROCAPE), Recife, PE, Brazil.; 2 Universidade de Pernambuco (UPE), Recife, PE, Brazil.; 3 Nucleus of Postgraduate and Research in Health Sciences of Faculdade de Ciências Médicas de Pernambuco/Instituto de Ciências Biológicas (FCM/ICB), Recife, PE, Brazil.

**Keywords:** Meta-Analysis, Drug-Eluting Stents, Coronary Artery Bypass, Stents, Percutaneous Coronary Intervention

## Abstract

**Objective:**

To compare the safety and efficacy of coronary artery bypass grafting (CABG) with percutaneous coronary intervention (PCI) using drug-eluting stents (DES) in patients with unprotected left main coronary artery (ULMCA) disease.

**Methods:**

MEDLINE, EMBASE, CENTRAL/CCTR, SciELO, LILACS, Google Scholar and reference lists of relevant articles were searched for clinical studies that reported outcomes at 1-year follow-up after PCI with DES and CABG for the treatment of ULMCA stenosis. Five studies fulfilled our eligibility criteria and they included a total of 4.595 patients (2.298 for CABG and 2.297 for PCI with DES).

**Results:**

At 1-year follow-up, there was no significant difference between CABG and DES groups concerning the risk for death (risk ratio [RR] 0.973, *P*=0.830), myocardial infarction (RR 0.694, *P*=0.148), stroke (RR 1.224, *P*=0.598), and major adverse cerebrovascular and cardiovascular events (RR 0.948, *P*=0.680). The risk for target vessel revascularization (TVR) was significantly lower in the CABG group compared to the DES group (RR 0.583, *P*<0.001). It was observed no publication bias regarding the outcomes, but only the outcome TVR was free from substantial statistical heterogeneity of the effects. In the meta-regression, there was evidence that the factor "female gender" modulated the effect regarding myocardial infarction rates, favoring the CABG strategy.

**Conclusion:**

CABG surgery remains the best option of treatment for patients with ULMCA disease, with lower TVR rates.

**Table:** 

Abbreviations, acronyms & symbols				
BMS	= Bare-metal stent		MeSH	= Medical Subject Heading
CABG	= Coronary artery bypass grafting		MI	= Myocardial infarction
CENTRAL/CCTR	= Cochrane Central Register of Controlled Trials		NR	= Non-reported
CI	= Confidence interval		OR	= Odds ratio
CK-MB	= Creatine kinase-MB		PCI	= Percutaneous coronary intervention
DS	= Diameter stenosis		PCI-DES	= Percutaneous coronary intervention/Drug-eluting stent
FFR	= Fractional flow reserve		PICOS	= Population, Intervention, Comparison, Outcome and Study Design
HR	= Hazard ratio		PRISMA	= Preferred Reporting Items for Systematic Reviews and Meta-Analyses
IVUS MLA	= Intravascular ultrasound minimal lumen area		RCTs	= Randomized controlled trials
JACC	= Journal of the American College of Cardiology		RR	= Risk ratio
LAD	= Left anterior descending		SciELO	= Scientific Electronic Library Online
LCX	= Left circumflex artery		SD	= Standard deviation
LILACS	= Latin American and Caribbean Health Sciences Literature		SE	= Standard error
LM	= Left main		TVR	= Target vessel revascularization
MACCE	= Major adverse cerebrovascular and cardiovascular events		ULMCA	= Unprotected left main coronary artery

## INTRODUCTION

### Rationale

The current international revascularization guidelines recommend revascularization of unprotected left main coronary artery (ULMCA) with coronary artery bypass grafting (CABG) or percutaneous coronary intervention (PCI) in subjects with low (<23: class I, recommendation for CABG or PCI - level of evidence B) and intermediate (23-32: class I for CABG and class IIa for PCI - level of evidence B) SYNTAX scores. The same guidelines recommend against revascularization with PCI of ULMCA disease with high SYNTAX scores (≥33: class I for CABG and class III for PCI - level of evidence B)^[1]^. Capodanno et al.^[2]^ published a meta-analysis of randomized controlled trials (RCTs) and suggested, boldly, that "based on their study, revision of the guidelines regarding left main PCI is warranted, raising the level of evidence of current recommendations from B to A". Although some RCTs have suggested that PCI with drug-eluting stent (DES) could be a non-inferior strategy which might be used safely^[3,4]^, sample sizes were not so large (and some conclusions may have been affected by this factor). Recently, the trials EXCEL^[5]^ and NOBLE^[6]^ were published, which led us to revisit the literature and carry out a new meta-analysis.

### Objective

We performed a meta-analysis of RCTs to compare CABG to PCI with DES for the treatment of patients with ULMCA disease, according to the Preferred Reporting Items for Systematic Reviews and Meta-Analyses (PRISMA) statement^[7]^.

## METHODS

### Eligibility Criteria

Using Population, Intervention, Comparison, Outcome and Study Design (PICOS) strategy, studies were considered eligible if: (1) the population comprised patients with ULMCA disease; (2) there was compared efficacy between CABG and PCI with DES; (3) the studied outcomes have included death, myocardial infarction (MI), stroke, target vessel revascularization (TVR) or major adverse cerebrovascular and cardiovascular events (MACCE); (4) there was a follow-up of at least 12 months. There was no restriction on language.

### Information Sources

The following databases were used (until December 2016): MEDLINE, EMBASE, Cochrane Central Register of Controlled Trials (CENTRAL/CCTR), ClinicalTrials.gov, Scientific Electronic Library Online (SciELO), Latin American and Caribbean Health Sciences Literature (LILACS), Google Scholar, and reference lists of relevant articles.

### Search

We conducted the search using Medical Subject Heading (MeSH) terms: ["coronary artery bypass graft" OR "coronary artery bypass grafting" OR "coronary artery bypass surgery" OR "coronary bypass surgery" OR "coronary artery bypass graft surgery" OR "coronary artery bypass" OR "coronary bypass"] AND ["drug-eluting stent" OR "sirolimus-eluting stent" OR "paclitaxel-eluting stent" OR "everolimus-eluting stent" OR "biolimus-eluting stent"] AND ["unprotected left main" OR "left main stenting" OR "left main coronary artery disease" OR "left main PCI" OR "unprotected left main coronary artery" OR "left main stenosis" OR "left main coronary artery stenting" OR "unprotected left main stenting"].

### Study Selection

The following steps were taken: (1) identification of titles of records through database searching; (2) removal of duplicates; (3) screening and selection of abstracts; (4) assessment for eligibility through full-text articles; (5) final inclusion in the study.

One reviewer followed the steps 1 to 3. Two independent reviewers followed the step 4 and selected studies. Inclusion or exclusion of studies was decided unanimously. When there was disagreement, a third reviewer took the final decision.

### Data Items

The primary endpoint was the risk ratio (RR) for mortality after PCI-DES or CABG, up to 12 months. Secondary endpoints were the RR for MI, stroke, TVR after the procedure, and MACCE (composite endpoint of death, MI, stroke or TVR).

### Data Collection Process

Two independent reviewers extracted the data. When there was disagreement about it, a third reviewer (the first author) checked the data and made the final decision. From each study, we extracted patients' characteristics, study design, and outcomes at 1 year after treatment of ULMCA stenosis. Alternatively, probabilities of mortality or MACCE were estimated from published Kaplan-Meier survival curves. When it was possible, we also extracted TVR from the total MACCE events and reported this outcome as a separate measure. When MACCE had not been reported, we calculated it using the events of death, MI, stroke and TVR, and reported this outcome as a separate measure.

### Risk of Bias in Individual Studies

Included studies were assessed for the following characteristics: sequence generation (randomization); allocation concealment (selection bias); blinding of participants and personnel (performance bias); blinding of outcome assessors (detection bias) and incomplete outcome data addressed (attrition bias)^[8]^.

Taking these characteristics into account, the papers were classified in A (low risk of bias), B (moderate risk of bias), C (high risk of bias) or D (unclear). Two independent reviewers evaluated the risk of bias. Agreement between the two reviewers was assessed with Kappa statistics for full-text screening and rating of relevance and risk of bias. When there was disagreement about risk of bias, a third reviewer checked the data and made the final decision.

### Summary Measures

The principal summary measures were RRs with 95% confidence interval (CI) and *P* values (statistically significant when <0.05). The meta-analysis was completed using the software Comprehensive Meta-Analysis version 2 (Biostat Inc., Englewood, NJ, USA).

### Synthesis of Results

Forest plots were generated for graphical presentations for clinical outcomes and we have performed the I-squared test and Chi statistics for assessment of heterogeneity across the studies^[9]^. Each study was summarized by the RR for PCI-DES compared to CABG. The RRs were combined across studies using DerSimonian-Laird random effects model^[10]^, weighted by number of events in each study.

### Risk of Bias Across Studies

To assess publication bias, a funnel plot was generated (for each outcome), being statistically evaluated by Begg and Mazumdar's test^[11]^ and Egger's test^[12]^.

### Sensitivity Analysis

We investigated the influence of a single study on the overall effect - by sequentially removing one study - to test the robustness of the main results, so we could verify whether any study had an excessive influence on the overall results or not.

### Meta-regression Analysis

Meta-regression analyses were performed to determine if the effects of CABG were modulated by pre-specified factors. Meta-regression graphs describe the effect of CABG on the outcome (plotted as a log RR on the y-axis) as a function of a given factor (plotted as a mean or proportion of that factor on the x-axis). Meta-regression coefficients show the estimated increase in log RR per unit increase in the covariate. Since log RR > 0 corresponds to RR > 1 and log RR < 0 corresponds to RR < 1, a negative coefficient will indicate that when a given factor increases, the OR decreases.

The predetermined modulating factors to be examined were: age (mean), female gender (%), diabetes (%), smoke (%), hypertension (%), SYNTAX score (mean) and distal left main lesion (%). This choice was made based on the factors that could recognizably modulate the summary measures when it comes to ULMCA disease. For studies reporting interquartile ranges, the mean was estimated according to the formula [minimum+maximum+2(median)]/4 and the standard deviation (SD) was calculated based on the formula (maximum-minimum)/6^[13]^.

## RESULTS

### Study Selection

**Fig. 1:**
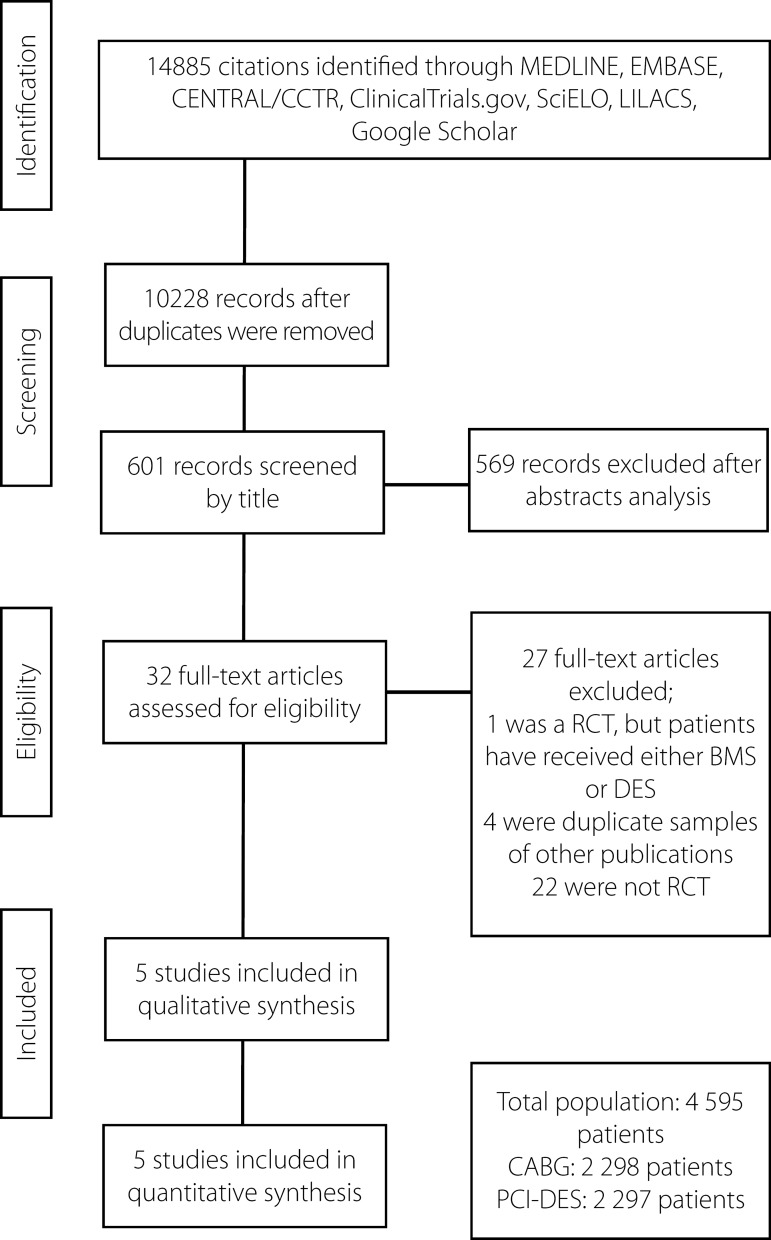
Flow diagram of studies included in data search. BMS=bare-metal stents; CABG=coronary artery bypass grafting; CENTRAL/CCTR=Cochrane Central Register of Controlled Trials; LILACS=Latin American and Caribbean Health Sciences Literature; PCI-DES=percutaneous coronary intervention/drug-eluting stent; RCT=randomized controlled trial; SciELO=Scientific Electronic Library Online

A total of 14.885 citations were identified, of which 32 studies seemed to be potentially relevant and were retrieved as full-texts. Five publications fulfilled our eligibility criteria^[3-6,14]^. Interobserver reliability of study relevance was excellent (Kappa=0.83). Agreement for decisions related to study validity was very good (Kappa=0.80). The search strategy can be seen in Figure 1.

### Study Characteristics

Characteristics of each study are shown in Table 1. A total of 4.595 patients were studied, with 2.298 receiving CABG and 2.297 receiving PCI with DES, during the years of 2003 to 2016. Two studies^[3,14]^ mostly used Cypher stent (sirolimus), one^[4]^ used Taxus stent (paclitaxel), one^[5]^ used Xience stent (everolimus) and one^[6]^ predominantly used Biomatrix Flex stent (biolimus; the latter was the recommended study stent but other "Conformité Européene-marked" DES could be used at the operators' discretion). The overall internal validity was moderate and is illustrated in Table 2.

**Table 1:** Study characteristics.

Study	PCI (n)	DES	Complete revascu-larization with PCI (%)	CABG (n)	LIMA to LAD (%)	Off-pump (%)	Complete revascu-larization with CABG (%)	Age (PCI/CABG) (years/mean)	Diabetes mellitus (PCI/CABG) (%)	SYNTAX score (PCI/CABG) (mean)	EuroSCORE (PCI/CABG) (mean)	Years
EXCEL trial^[5]^	948	Xience (100%)	NR	957	98.8	29	NR	66/66	30/28	21/21	NR	2010-2016
NOBLE trial^[6]^	592	Biomatrix Flex (predominantly)	92	592	93	16	NR	66/66	15/15	23/22	2/2	2008-2015
PRECOMBAT trial^[3]^	300	Cypher (100%)	68	300	93.6	63.8	70	62/63	34/30	24/26	2.6/2.8	2004-2009
SYNTAX trial^[4]^	357	Taxus (100%)	65	348	97	NR	73	66/65	24/26	30/30	3.9/3.9	2005-2007
Boudriot et al.^[14]^2011	100	Cypher (98%)Taxus (2%)	98	101	99	46	97	66/69	40/30	24/23	2.4/2.6	2003-2009

Biomatrix Flex: Biolimus-eluting stent; Cypher: sirolimus-eluting stent; Taxus: paclitaxel-eluting stent; Xience: everolimus-eluting stent. CABG=coronary artery bypass grafting; DES=drug-eluting stent; LAD=left anterior descending artery; LIMA=left internal mammary artery; PCI=percutaneous coronary intervention; NR = non-reported

**Table 2:** Analysis of risk of bias – internal validity.

Study	Selection bias	Performance bias	Attrition bias	Detection bias	Multivariate adjustment for possible confounders
EXCEL trial^[5]^	A	C	A	A	Probably adequate
NOBLE trial^[6]^	A	C	A	A	Probably adequate
PRECOMBAT trial^[3]^	A	C	A	A	Probably adequate
SYNTAX trial^[4]^	A	C	A	D	Probably adequate
Boudriot et al.^[14]^2011	A	C	A	A	Probably adequate

A=risk of bias is low; B=risk of bias is moderate; C=risk of bias is high; D=unclear to determine

### Synthesis of Results

**Fig. 2:**
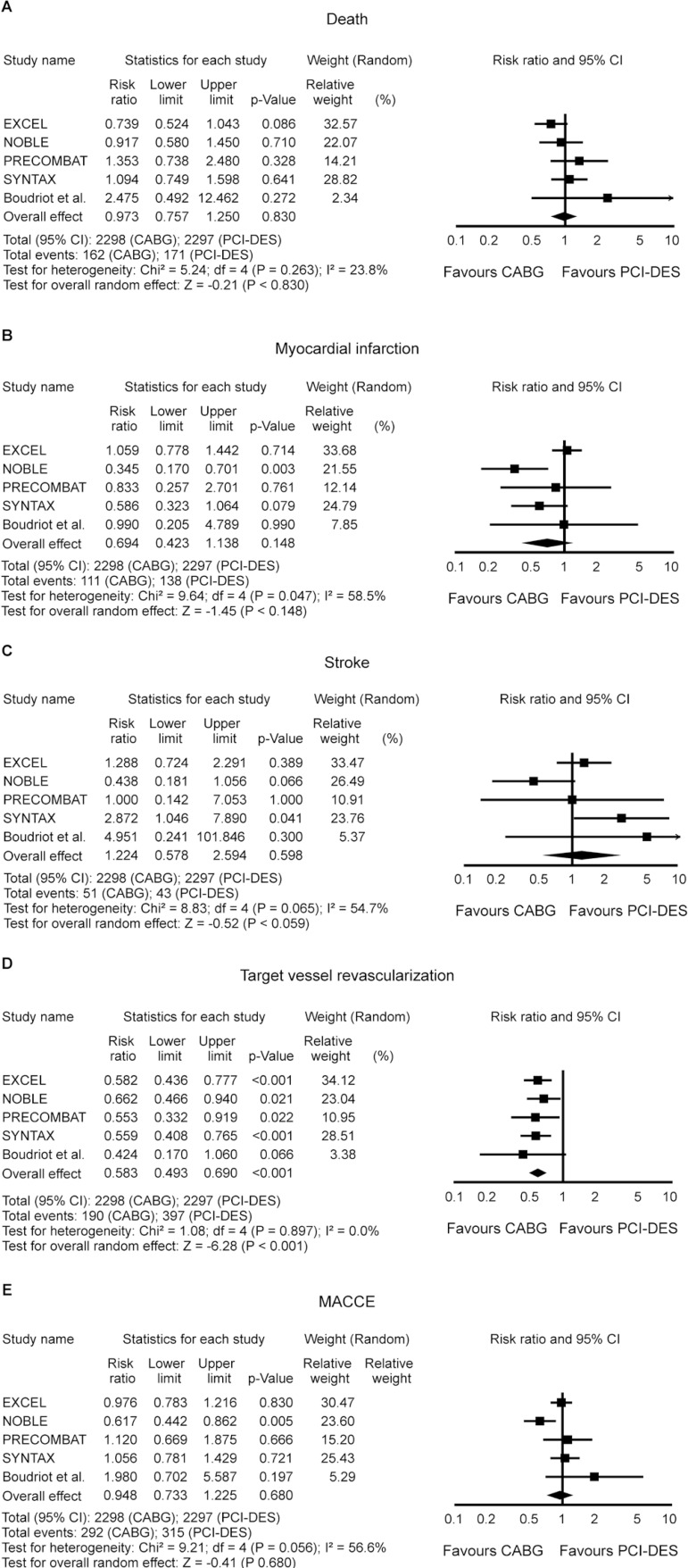
Risk ratio (RR) and conclusions plot of death, myocardial infarction (MI), stroke, target vessel revascularization (TVR), and major adverse cerebrovascular and cardiovascular events (MACCE) associated with coronary artery bypass grafting (CABG) vs. percutaneous coronary intervention/drug-eluting stent (PCI-DES). CI=Confidence interval

The RR for death in the CABG group compared with that in the PCI-DES group in each study, at 1-year time point, is reported in Figure 2A. There was evidence of moderate heterogeneity of treatment effect among the studies for death. The overall RR (95% CI) of death showed no difference between CABG and PCI-DES at 1-year (random effect model: RR 0.973, *P*=0.830).

The RR for MI in the CABG group compared with that in the PCI-DES group in each study, at 1-year time point, is reported in Figure 2B. There was evidence of substantial heterogeneity of treatment effect among the studies for MI. The overall RR (95% CI) for MI showed no difference between CABG and PCI-DES at 1-year (random effect model: RR 0.694, *P*=0.148).

The RR for stroke in the CABG group compared with that in the PCI-DES group in each study, at 1-year time point, is reported in Figure 2C. There was evidence of substantial heterogeneity of treatment effect among the studies for stroke. The overall RR (95% CI) for stroke showed no difference between CABG and PCI-DES at 1-year (random effect model: RR 1.224, *P*=0.598).

The RR for TVR in the CABG group compared with that in the PCI-DES group in each study, at 1-year time point, is reported in Figure 2D. There was no evidence of heterogeneity of treatment effect among the studies for TVR. The overall RR (95% CI) for TVR showed a statistically significant difference between CABG and PCI-DES at 1-year, favoring CABG (random effect model: RR 0.583, *P*<0.001).

The RR for MACCE in the CABG group compared with that in the PCI-DES group in each study, at 1-year time point, is reported in Figure 2E. There was evidence of substantial heterogeneity of treatment effect among the studies for MACCE. The overall RR (95% CI) for MACCE showed no difference between CABG and PCI-DES at 1-year (random effect model: RR 0.948, *P*=0.680).

### Risk of Bias Across Studies

**Fig. 3:**
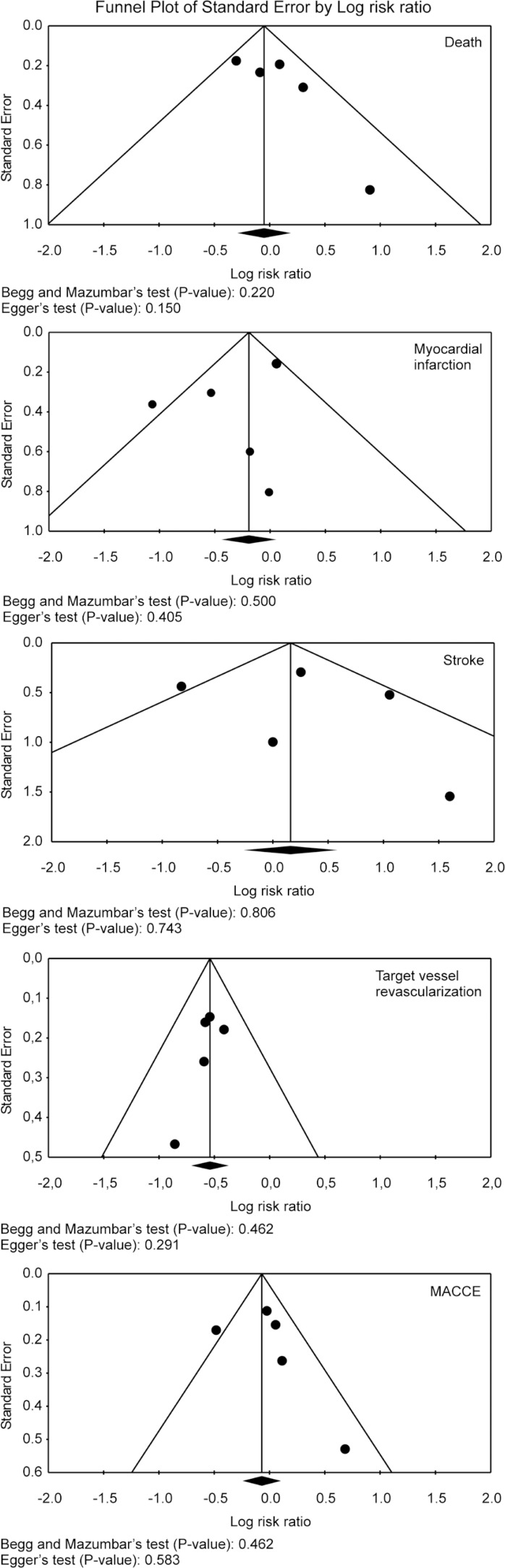
Publication bias analysis by funnel plot graphic. MACCE=Major adverse cerebrovascular and cardiovascular events

Funnel plot analysis (Figure 3) disclosed no asymmetry around the axis for the treatment effect when the outcomes were analyzed, which means we probably have no publication bias related to these outcomes.

### Sensitivity Analysis

**Fig. 4:**
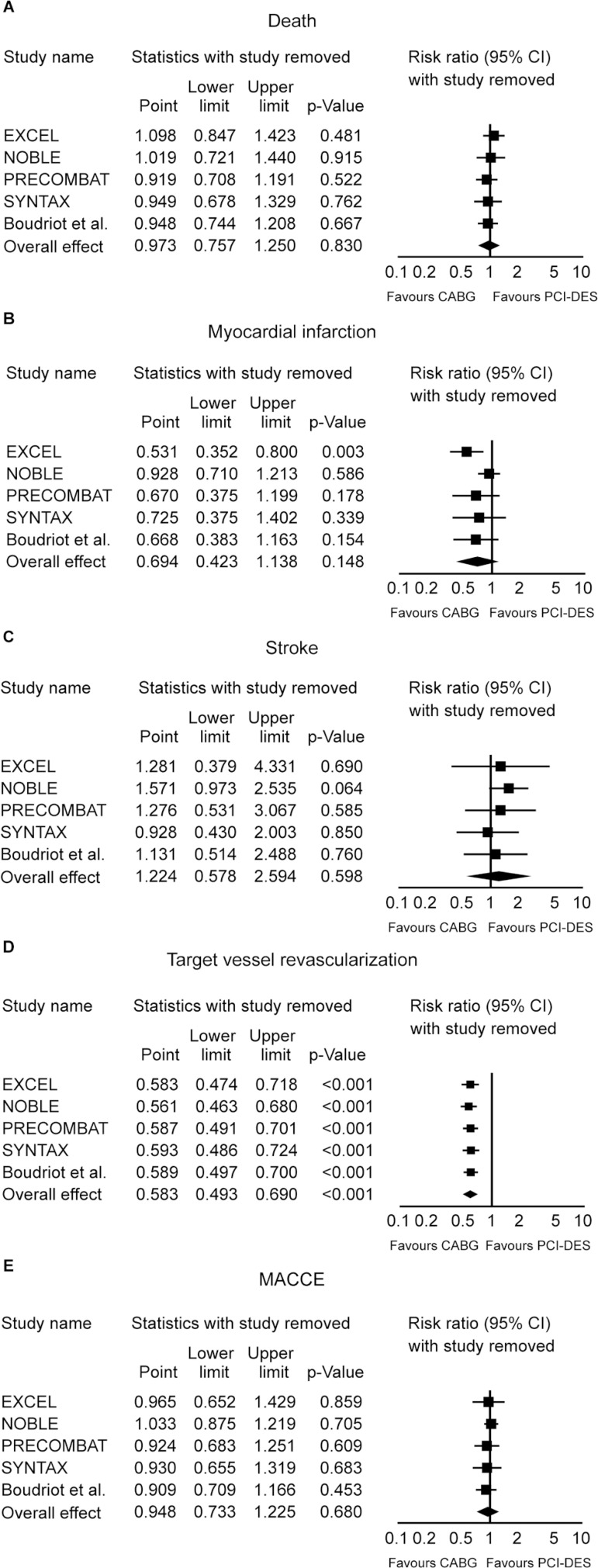
Sensitivity analyses (one study removed). CABG=coronary artery bypass grafting; CI=confidence interval; MACCE=major adverse cerebrovascular and cardiovascular events; PCI-DES=percutaneous coronary intervention/drug-eluting stent

Sensitivity analyses performed by removing each single study from the meta-analysis to determine the influence of an individual data set to the pooled RR showed that the EXCEL trial caused a major change in direction/magnitude of statistical findings regarding the outcome MI (Figure 4B). When this study was removed from the analysis, a statistically significant difference in favor of CABG appeared, which means that this study actually had an excessive influence on the overall results concerning the outcome MI, favoring excessively the PCI-DES when it was included.

### Meta-regression Analysis

**Fig. 5:**
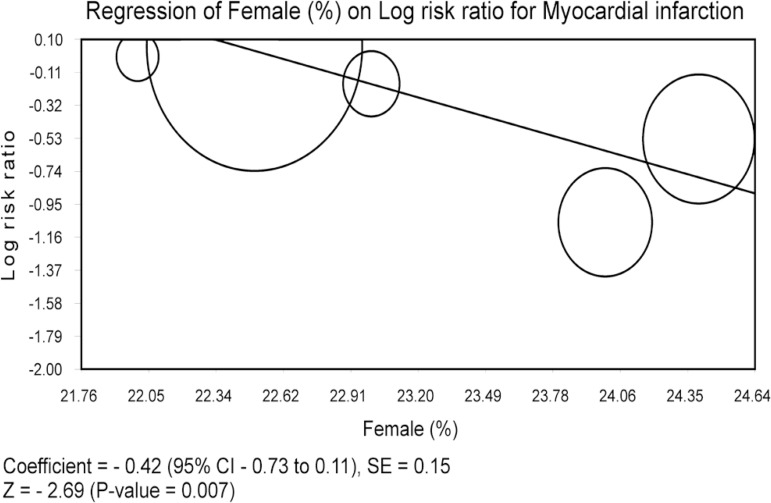
Meta-regression analysis. CI=confidence interval; SE=standard error

Meta-regression coefficients were not statistically significant for age, diabetes, smoke, hypertension, SYNTAX score or distal left main lesion, which means that none of these evaluated factors had any modulation influence on the final effect, regarding death, MI, stroke, TVR or MACCE. When we analyzed the outcome "MI" and the pre-determined factor "female gender", it was observed a statistically significant coefficient for proportion of female patients and RR for MI (coefficient -0.42; 95% CI -0.73 to 0.11; *P*=0.007; Figure 5). We can observe that the greater the proportion of female patients, the lower the RR for MI in the group CABG in comparison to PCI-DES group.

## DISCUSSION

### Summary of Evidence

The results of this meta-analysis demonstrate that PCI with DES for ULMCA disease presents a significantly higher risk for TVR at 1-year follow-up in comparison to CABG, being this outcome under no influence of statistical heterogeneity or publication bias. Although there was no difference in the risk for death, MI, stroke and MACCE, these summary measures were underpowered by heterogeneity of the effects.

We've also observed that the EXCEL trial had had a major influence on the overall results regarding the outcome MI, favoring excessively the group PCI-DES. Interestingly, as evidenced by the meta-regression analysis, the gender seems to play a certain role in the results, since we've detected that when there were more women in the group CABG, more beneficial was this strategy in comparison to PCI with-DES.

### Considerations

The length of follow-up considered for this study may have been too short (1 year) to truly detect differences between the treatment groups. CABG is associated with a higher early mortality rate from perioperative complications, and it is possible that with a longer follow-up, CABG patients may have an improved survival rate compared to patients undergoing PCI with DES. Curiously, we've observed no statistically significant difference regarding the outcome stroke, although we had expected a higher risk in the CABG group. The long-term durability of PCI *vs.* CABG remains undetermined and will require studies with longer follow-ups to produce a robust meta-analysis of long-term results. Up to now, only SYNTAX, PRECOMBAT and NOBLE trials published their 5-year follow-ups.

Regarding the major influence on the overall rate of MI exerted by the EXCEL trial, favoring excessively the group PCI-DES, we must bear in mind that this study excluded patients with SYNTAX score ≥33. On the other hand, the NOBLE trial has enrolled patients with ostium, mid-shaft and/or bifurcation and with no more than three additional non-complex PCI lesions, defined as length <25 mm, non-chronic total occlusion, non-two-stent bifurcation, non-calcified and non-tortuous coronary lesions.

The gender seemed to play a certain role in the results. Intriguingly, there are studies showing that women experience higher short-term and long-term mortality rates after PCI compared to men^[15]^ and showing that women have worse longterm outcomes after CABG than men^[16]^. To sum up, women tend to benefit less from both invasive strategies. Nevertheless, when it comes to comparing CABG with PCI-DES, it seems that the former presents better results, since studies showed that CABG may provide the greatest benefit to patients who have most extensive heart disease^[17,18]^, maybe because women (compared with men) are, on average, older and more likely to have diabetes and hypertension and to present for surgery with urgent/ emergent status^[16]^.

One of the limitations is the heterogeneity of the strategies across the studies. Among PCI strategies, some studies used either only sirolimus-stent or only paclitaxel-stents or predominantly biolimus-stents or only everolimus-stents, and some mixed drug-eluting stents, etc. Campos et al.^[19]^ underlined some differences about how the Heart Team assessed the ULMCA as being significant in the studies. For example, the NOBLE trial adopted as significant an ULMCA with a visually assessed DS>50% or a fractional flow reserve (FFR) <0.80. The EXCEL trial defined significant ULMCA as one of the following: DS≥70% (visually estimated) or DS≥50% but <70% (requiring non-invasive or invasive [FFR ≤0.80] evidence of ischaemia or intravascular ultrasound minimal lumen area [IVUS MLA] ≤6.0 mm^2^). Additionally, the EXCEL trial has enrolled patients with a left main equivalent disease, defined as bifurcation disease, with both the ostial left anterior descending (LAD) artery and ostial left circumflex artery (LCX) stenoses having ≥70% DS. If one or both ostial LAD and ostial LCX stenoses are ≥50% and <70% stenotic by visual estimation, then this(ese) lesion(s) is(are) demonstrated to be significant either by non-invasive or invasive (FFR ≤0.80) evidence of ischaemia in its myocardial distribution or IVUS MLA ≤4.0 mm^2^. By protocol, in EXCEL, FFR was the preferred strategy to stratify lesion significance. Among CABG strategies, there is variability in rates of use of internal thoracic artery, use of cardiopulmonary bypass (on-pump *vs.* off-pump CABG), etc. And between both strategies, an important aspect to consider is the rate of complete revascularization (that was not reported adequately in 2 studies^[5,6]^, but in the 2 studies^[3,4]^ that reported these rates, both arms presented high rates of incomplete revascularization, reaching around 30%), which reflects in the outcomes.

Curiously, the publication of EXCEL and NOBLE trials, both studying the same issue and emerging with different results, may have confused some cardiologists and cardiovascular surgeons. In the EXCEL study, investigators randomized 948 patients with ULMCA disease to PCI with Xience and 957 patients to CABG surgery. The primary endpoint-a composite that included allcause mortality, stroke, or MI at 3 years-occurred in 15.4% of patients treated with PCI and 14.7% of patients treated with CABG (*P*=0.02 for non-inferiority). The researchers also analyzed data at the 30^th^ day, a secondary endpoint. The rate of death, stroke or MI was significantly higher among the CABG-treated patients (4.9% *vs.* 7.9%; HR 0.61; 95% CI 0.42-0.88), and this difference was driven by a considerably increased risk of MI (3.9% *vs.* 6.2%; HR 0.63; 95% CI 0.42-0.95). In particular, there was a significantly increased risk of "large" periprocedural MI with CABG, which was defined as CK-MB more than 10 times the upper limit of normal (or 5 times the upper limit of normal plus other evidence of MI). In the NOBLE trial, 1,201 patients with ULMCA disease were randomized to PCI or CABG. The 5-year estimate of MACCE-a composite of all-cause mortality, nonprocedural MI, any repeat coronary revascularization, and stroke-occurred in 29% of patients treated with PCI and 19% of patients who underwent CABG, a difference that exceeded the limit for non-inferiority (*P*=0.007 for superiority). Therefore, the results of these studies are clearly conflicting.

There are inherent limitations in meta-analyses, including the use of cumulative data from summary estimates. Patients' data were gathered from published data, not from individual patient follow-up. Access to individual patient's data would have enabled us to conduct further subgroup analysis and propensity analysis to account for differences between the treatment groups. This meta-analysis included only data from randomized studies, which do not reflect the "real world" but, on the other hand, are less limited by publication bias, treatment bias, confounders, and a certain tendency to overestimate the treatment effects observed in observational studies, since patients' selection alters the outcomes and thus makes non-randomized studies less robust.

A final limitation is the absence of adequate published comparative data for the third therapeutic option, medical therapy. PCI with DES has not been compared yet with medical therapy alone when we consider ULMCA disease, but CABG has been shown to be superior to medical therapy in this set.

## CONCLUSION

We found evidence that argues against the so-called "non-inferiority" of PCI with DES in comparison to CABG surgery and against the idea that PCI can be considered a reasonable choice in elective cases (not mentioning prohibitive risk patients, acute patients, and those who reject surgery). Given that, although the rates of death, MI, stroke, and MACCE between both strategies were not statistically different (remembering the heterogeneity related to these outcomes), the need of new procedures were clearly lower in patients treated with CABG surgery. However, careful analysis of the data shows that no definite conclusion can be drawn from the evidence available due to the heterogeneity of studies regarding the outcomes, heterogeneity of strategies (different drug-eluting stents, different ways to perform surgery, etc), and heterogeneity of coronary lesions complexity.

Needless to say, we are living in a changing world. It is incredible how fast the winds of change blow through the medical literature. Nowadays, an author publishes a meta-analysis boldly recommending changing the level of evidence of PCI-DES in ULMCA patients in the Journal of the American College of Cardiology (JACC), a high-quality journal, and then, after new evidence came out, the same author changes his/her stance on this issue one more time. New data will come when we have the 5-year results of these trials, when the scientific community will be enlightened with stronger and definitive results. Disagreeing with other authors, we conclude that "based on our study, revision of the guidelines regarding left main PCI with DES must be viewed with caution, and we still do not have enough evidence that makes the level of evidence of current recommendations raises from B to A".

**Table:** 

Authors' roles & responsibilities	
MPBOS	Conception and study design; data management; manuscript redaction or critical review of its content; final manuscript approval
AFS	Substantial contributions to the conception or design of the work; or the acquisition, analysis, or interpretation of data for the work; final manuscript approval
RGAM	Substantial contributions to the conception or design of the work; or the acquisition, analysis, or interpretation of data for the work; final manuscript approval
MLA	Substantial contributions to the conception or design of the work; or the acquisition, analysis, or interpretation of data for the work; final manuscript approval
AMM	Conception and study design; data management; manuscript redaction or critical review of its content; final manuscript approval
FPVS	Conception and study design; data management; manuscript redaction or critical review of its content; final manuscript approval
RCL	Conception and study design; data management; manuscript redaction or critical review of its content; final manuscript approval
